# Editorial: Interaction of microbiota and metabolomic disorders

**DOI:** 10.3389/fcimb.2025.1597214

**Published:** 2025-04-29

**Authors:** Jorge A. M. Pereira

**Affiliations:** Centro de Química da Madeira (CQM), Universidade da Madeira, Funchal, Portugal

**Keywords:** microbiota, metabolism, microbial-metabolites interaction, metabolic disorder, disease

The interplay between the human microbiome and metabolic abnormalities in health and disease has recently become a prominent research area. The term ‘microbiota’ encompasses the vast array of microorganisms that inhabit the human body, predominantly within the gastrointestinal tract. These minute life forms are integral to maintaining optimal health, facilitating nutrient breakdown, synthesising essential vitamins, and influencing immune defences (Scotti et al.).

Metabolomic disorders are defined by irregular concentrations or functions of metabolites. Disruptions in metabolite balance have been associated with various ailments, including obesity, diabetes, cardiovascular conditions, bone metabolic issues, atypical tumour metabolism, and even psychological disorders (Gu et al., Muller at al., Szczerbinski et al., Suvitaival et al.). Scientists are currently exploring this field to elucidate the underlying processes and to identify potential diagnostic or predictive biomarkers for these conditions. Studies have demonstrated that the composition and function of intestinal microbiota can affect the generation and processing of diverse metabolites. For example, specific bacteria can generate short-chain fatty acids (SCFAs) through the breakdown of dietary fibre. These SCFAs have been linked to numerous health advantages, such as anti-inflammatory properties and the regulation of energy metabolism (Ghosh and Pramanik).

In this context, this Research Topic explored the interplay between microbiota and metabolomic disorders. Different reports contributed to elucidate the impact of microbiota on various metabolic disorders, including obesity (Li et al., Song et al.), diabetes, cardiovascular ailments (Hu et al.), inflammatory responses (Jiang et al.), gastrointestinal conditions (Wang et al.), bone metabolism and osteoporosis (Wu et al., Hao et al.), thyroid disease (Fang and Ning), and infertility (Wang et al.). By delving into this relationship, this Research Topic sheds light on prospective therapeutic methods or interventions that could manipulate microbiota to combat or mitigate metabolomic disorders.

Hu et al. research explored how gut microbiota and metabolome impact the lipid-lowering effects of statins in Chinese patients with coronary heart disease and hypercholesterolemia. This study aimed to identify biomarkers to evaluate differences in statin effectiveness. Metabolomic analysis revealed that the levels of chenodeoxycholic acid-3-b-D-glucuronide, 1-methylnicotinamide, and acetoacetate were significantly elevated in stool samples from the group with an increased risk of coronary heart disease compared to the group with lower risk. Li et al. analysed primary host gene-microbe networks in the caecum tissues of rabbits that became obese owing to a high-fat diet. A metagenomic study revealed changes in the abundance of bacterial species. A link was found between gene activation and specific bacterial species, indicating microbial and gene interactions in the caecum of obese rabbits. This research lays the groundwork for future investigations of intestinal interventions for human obesity. Song et al. conducted a comprehensive analysis of the gut microbiome and its metabolites in mice with either a knockout or overexpression of the angiotensin-converting enzyme 2 (ACE2). Their research points to a significant link between ACE2 status and the profiles of the gut microbiome and metabolome, revealing a new mechanism by which ACE2 positively influences the energy balance. Wang et al. investigated the interaction between intestinal microbiota and hypertensive disorders of pregnancy (HDP), establishing a link between HDP and immune regulation of the gut microbiota. Technologies such as macrogenomics and metabolomics are expected to enhance precision medicine and advanced health management, providing additional data necessary to unveil the mechanisms involved in these processes. Moreover, enhanced medical strategies for pregnancy will benefit mothers and children with HDPs, and testing gut bacteria will improve the diagnosis and treatment of this condition.

Wu et al. performed a comprehensive bibliometric analysis of global research on the interplay between the gastrointestinal microbiota and bone metabolism, offering valuable insights for further investigations within this domain. This study identified molecular biology, immunology, and clinical medicine as future research hotspots, and highlighted the therapeutic potential of probiotics and prebiotics in modulating bone metabolism, particularly in conditions such as osteoporosis.

Related to this Research Topic, Hao et al. examined the impact of gut microbiota (GM) on osteoporosis pathogenesis (OP) and management, providing a comprehensive overview of the GM-bone axis. The authors outlined the risk factors and pathogenesis of OP, explored GM diversity and functional changes in OP, and examined evidence linking GM alterations with variations in bone mineral density and fracture risk. They also identified future research directions and challenges in translating the findings into clinical practice. Among these, well-designed clinical trials evaluating the efficacy, safety, and long-term effects of GM-targeted interventions, such as probiotics and prebiotics, on improving bone health outcomes are particularly relevant. In addition, the incorporation of individual variability in GM composition and lifestyle factors into personalised medicine approaches holds promise for tailoring OP management strategies.

Jiang et al. explored the associations between gut microbiota and inflammatory response and early haematoma expansion (HE) in intracerebral haemorrhage (ICH), revealing significant variations in the diversity and even distribution of microorganisms in the different groups analysed, as well as evidence for the association between the increase in the number of gram-negative pro-inflammatory bacteria and a decline in the level of probiotics and systemic inflammation.

Fang and Ning reviewed recent advances in gut microbiota and thyroid disease and reported significant variations in the gut composition of several bacteria. These alterations are implicated in the development and progression of thyroid diseases by affecting metabolic pathways crucial for immune regulation and thyroid hormone homeostasis. Evidence suggests that probiotic adjunct therapy can modulate gut microbiota and improve thyroid function and patient outcomes.

Finally, Wang et al. explored the link between the gut microbiota and diseases related to infertility, aiming to identify new biomarkers and possible treatments. They examined the developmental processes connecting gut microbiota with polycystic ovary syndrome, endometriosis, and premature ovarian failure from a fresh perspective, offering innovative ideas for diagnosing and treating female infertility conditions and providing specific reference values for eugenics.

This editorial highlights the interplay between the gut microbiota and metabolic disorders. Different studies have shown that gut microbiota alterations are linked to conditions such as obesity, hypertension, osteoporosis pathogenesis (OP), management, and inflammatory responses, opening avenues for personalised medicine based on microbiome profiles. The integration of macrogenomics and metabolomics will enhance our understanding of these interactions, leading to improved health-management strategies. These insights suggest a paradigm shift in the treatment of metabolic disorders through microbiota modulation, with future clinical trials being crucial for validating findings and developing therapeutic interventions.

